# Fatty Acid Desaturase Gene Polymorphisms and Metabolic Measures in Schizophrenia and Bipolar Patients Taking Antipsychotics

**DOI:** 10.1155/2013/596945

**Published:** 2013-12-21

**Authors:** Kyle J. Burghardt, Kristen N. Gardner, Joshua W. Johnson, Vicki L. Ellingrod

**Affiliations:** ^1^Department of Clinical Social and Administrative Sciences, University of Michigan College of Pharmacy, 428 Church Street, Ann Arbor, MI 48109, USA; ^2^University of North Carolina Eshelman School of Pharmacy, 301 Pharmacy Lane, Chapel Hill, NC 27599, USA; ^3^Department of Psychiatry, School of Medicine, University of Michigan, 1500 Medical Center Drive, Ann Arbor, MI 48109, USA

## Abstract

Atypical antipsychotics have become a common therapeutic option in both schizophrenia and bipolar disorder. However, these medications come with a high risk of metabolic side effects, particularly dyslipidemia and insulin resistance. Therefore, identification of patients who are at increased risk for metabolic side effects is of great importance. The genetics of fatty acid metabolism is one area of research that may help identify such patients. Therefore, in this present study, we aimed to determine the effect of one commonly studied genetic polymorphism from both fatty acid desaturase 1 (*FADS1*) and *FADS2* gene on a surrogate measure of insulin resistance and lipid levels in a metabolically high-risk population of patients largely exposed to atypical antipsychotics. This study used a cross-sectional design, fasting blood draws, and genetic analysis to investigate associations between polymorphisms, haplotypes, and metabolic measures. A total of 320 subjects with schizophrenia (*n* = 226) or bipolar disorder (*n* = 94) were included in this study. The mean age of the population was 42.5 years and 45% were male. A significant association between *FADS1* and *FADS2* haplotypes was found with insulin resistance while controlling for confounders. Further investigation is required to replicate this finding.

## 1. Background

The use of antipsychotics, particularly the atypical antipsychotics (AAPs), is considered the standard of care in schizophrenia symptom management and is becoming a common therapeutic choice in the management of bipolar disorder [1–3]. The fact that AAPs are commonly used in both of these populations may be due to the overlapping symptomatology that is seen as well as the genetic overlap that has been identified in several disease linkage studies [4–7]. Although many studies support the use of AAPs in the severely mentally ill, these medications come with a high risk of metabolic side effects. This risk requires careful monitoring and management as the cardiometabolic side effects have been shown to increase the cost of care, decrease adherence, and, most severely, have negative consequences on length and quality of life [8–11]. Therefore, investigation into lifestyle, diet, and genetic factors that may increase or attenuate the risk of metabolic side effects in patients taking AAPs is important and of high interest. One current line of research within the area of AAP metabolic side effects concentrates on fatty acid metabolism and its influence on metabolic measures.

Fatty acids (FAs) serve many important physiological functions including energy reserves, structural components of cell membranes, precursors of eicosanoids, and regulators of gene expression. The role FAs play in cell membranes is of particular interest as they influence translocation of glucose transporters and insulin receptor binding and signaling in addition to cell membrane fluidity and permeability. This indicates that FAs may play an important role in the development of insulin resistance and type 2 diabetes mellitus [12–14]. FA levels in the blood are determined by both dietary FA intake and to a larger extent endogenous FA metabolism. Endogenous FA metabolism is mediated by a series of elongation and desaturation steps controlled by two rate-limiting enzymes called delta-5 desaturase (D5D) and delta-6 desaturase (D6D). These enzymes, which are expressed at high levels in the liver, brain, heart, and lungs, are responsible for conversion of linoleic acid to n-6 polyunsaturated fatty acids (PUFA) and *α*-linolenic acid to n-3 PUFAs. Furthermore, it has been determined that D5D is encoded by the fatty acid desaturase 1 (*FADS1*) gene and D6D is encoded by the fatty acid desaturase 2 (*FADS2*) gene which are located on chromosome 11. Minor alleles of polymorphisms within the *FADS1/2* gene cluster are commonly associated with lower D5D and D6D activities and thus are used as a surrogate marker for desaturase activity [15]. Many studies in various populations have shown correlations with *FADS1* and/or *FADS2* polymorphisms and FA or lipid levels [16, 17]. Additionally, there is also a growing body of literature showing correlations between D5D and D6D activity, insulin resistance, and risk for type 2 diabetes mellitus. The two desaturases have an opposite effect on the risk of developing diabetes. Specifically, an increased D5D activity is associated with lower risk of developing diabetes, whereas increased D6D activity is associated with a higher risk. A few studies have indicated possible differences in FA desaturase activity and *FADS* gene expression due to the influence of antipsychotics; however this data primarily comes from animal populations and postmortem brain studies. Thus, to our knowledge no studies have investigated *FADS* genetic variants and metabolic biomarkers from the mental health population taking antipsychotics [18–23].

Therefore, in this present study, we aimed to determine the effect of one commonly studied genetic polymorphism (SNP) from both the *FADS1 *and *FADS2* gene on a surrogate measure of insulin resistance and lipid levels in a metabolically high-risk population of patients diagnosed with schizophrenia or bipolar disorder and largely exposed to AAPs. We also aimed to use a haplotype analysis to evaluate the combined effects of each gene's variant on insulin resistance and lipid levels.

## 2. Subjects and Methods

### 2.1. Study Population

Male and female participants were recruited from outpatient mental health clinics in the Southeastern Michigan area. Subjects were considered for inclusion if they met the following criteria: (1) aged 18–80 and diagnosed with schizophrenia, schizoaffective disorder, or bipolar disorder, (2) currently taking an antipsychotic, and (3) no medication changes for the previous 6 weeks. Subjects were excluded based on the following criteria: (1) having an active substance abuse or dependence diagnosis, (2) currently taking a medication for diabetes (to avoid bias in the insulin resistance measure), or (3) unwilling or unable to participate. The study was approved by the University of Michigan Institutional Review Board.

### 2.2. Clinical and Metabolic Measurements

Participants came to the University of Michigan Clinical Research Unit (MCRU) for a single visit. Study visits were completed in the morning, within 2 hours of the participants' usual wakening time. Participants were required to fast overnight for the visit. After obtaining an informed consent, participants underwent the structured clinical interview for DSM-IV-TR diagnoses (SCID) performed by a trained research assistant in order to confirm their psychiatric diagnosis. Psychiatric diagnoses were also confirmed by medical chart review when possible. Subjects were asked about basic demographic information (e.g., age, race, and gender), current medications (also confirmed by pharmacy records), and current or past cigarette smoking. A registered nurse took height and waist measurements along with a blood pressure measurement and a blood draw. Body mass index (BMI, kg/m^2^) was calculated from this information. The blood draw was collected for genetic analysis and fasting labs such as lipids (which included total cholesterol (TC), triglycerides (TG), high-density lipoproteins (HDL), and low-density lipoprotein (LDL)), blood glucose, and insulin measurements were measured. Insulin resistance was calculated using the homeostatic model assessment of insulin resistance [24] (HOMA-IR, [fasting insulin (*μ*IU/mL) × fasting glucose (mmol/L)]/22.5) which has been correlated to more invasive measures of insulin resistance [25–28].

### 2.3. Selection of SNPs and Genotyping Methods

Whole blood was used for DNA extraction using the salt precipitation method [29]. We aimed to conduct a candidate-gene study by using one polymorphism from both the *FADS1 *and *FADS2* genes. The variants, *FADS1* rs174537 and *FADS2* rs174570, were chosen based on a literature review in which these variants were associated with metabolic measures in various large cohorts with replicated results [17, 29–34]. The *FADS1* rs174537 (G/T) variant is located 14 kb upstream of the *FADS1* gene and has a minor allele frequency (MAF) of approximately 33% (from the 1000 genomes project) while the *FADS2* rs174570 (C/T) is located in intron 1 of the *FADS2* gene and has a MAF of approximately 24% (from the 1000 genomes project). Genotyping was completed by polymerase chain reaction followed by pyrosequencing [35] (specific assay conditions available upon request).

### 2.4. Haplotype Analyses

Linkage analysis between the *FADS1* and *FADS2* variants and evaluation of a haplotype block were conducted by using Haploview 4.2 [36]. Pairwise haplotypes were inferred using the PHASE 2.1 program and only haplotypes with a frequency >1% were used in statistical analysis [37, 38]. PHASE 2.1 uses a Bayesian statistical method for determining haplotypes from population data. The computational algorithms used in the PHASE 2.1 program have been shown to be superior compared to other commonly employed haplotype inference methods like the expectation-maximization (EM) algorithm.

### 2.5. Statistical Analysis

Statistical analyses were performed with JMP Pro 9.0 software (JMP, Version 9.0. SAS Institute Inc., Cary, NC, 1989–2012). Hardy-Weinberg equilibrium (HWE) was evaluated using Haploview 4.2. One-way analysis of variance (ANOVA) was used to assess differences in mean values of clinical and metabolic variables within psychiatric diagnosis and genetic variant groups (*FADS1 *SNP, *FADS2* SNP, and *FADS1/2* haplotypes) for continuous variables (age, BMI, blood pressure, lipid levels, glucose, insulin, and HOMA-IR). Chi-squared analysis was used to compare dichotomous variables (gender, race, AAP status, and smoking status) by psychiatric diagnosis and genetic variant groups. A two-tailed value of *P* < 0.05 was considered statistically significant for these tests. To examine our main hypothesis, the relationship between either SNP or haplotype variants and lipid or HOMA-IR, a regression model was constructed using the metabolic measures (TC, TG, HDL, LDL, or HOMA-IR) as the dependent variable and psychiatric diagnosis, age, race, gender, bmi, smoking status, and AAP status as the independent variables. In order to account for multiple testing and reduce the rate of false positives, a *Bonferroni* correction was applied for our regression analyses. Given tests were conducted for each SNP and haplotype for the four lipid values (TC, TG, LDL, and HDL) and HOMA-IR; only *P* values <0.003 were considered statistically significant (0.05/15 tests analyzed in regression). Results are expressed as means ± standard deviation (S.D.) or %.

## 3. Results

### 3.1. Study Population Characteristics

A total of 320 subjects with schizophrenia (*n* = 226) or bipolar disorder (*n* = 94) were included in this study. The mean age of the population was 42.5 years, with 45% male and 70% identifying themselves as Caucasian.  Table 1 represents the demographic and metabolic parameters of our study population. The schizophrenia spectrum and bipolar disorder cohorts were compared and significant differences were found for gender, race, current smokers, AAP use, and three lipid measures (TC, HDL, and LDL). Therefore, in our analyses of the combined cohort regarding our main hypotheses, psychiatric diagnosis was used as a confounder in order to account for differences between the two populations taking antipsychotics.

### 3.2. Genetic Distribution and Analysis for *FADS* Variants

**Figure 1:**
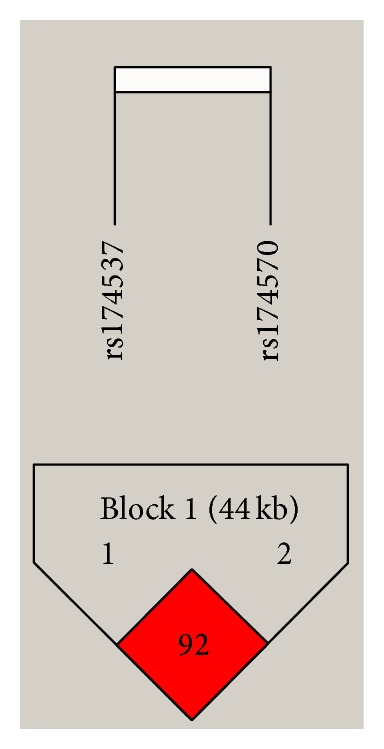
*FADS* haplotype block. Haplotype block analysis using the *four-gamete rule* for the *FADS1 *and *FADS2* SNPS investigated in this study.

The *FADS1* and *FADS2* variants used in this study satisfied HWE within the schizophrenia and bipolar populations as well as in the combined sample (all *P* > 0.1).  Table 2 illustrates the distribution of the variants within our population based on a dichotomized, dominant genetic model (e.g., wildtype = 0 and heterozygote/homozygote = 1). This model was used due to the low frequency of homozygote variants seen with the *FADS* variants as well as previous studies establishing this model as predictive of FA desaturase enzyme activity. Of note, two samples were unable to be genotyped for the *FADS1* variant due to sample fatigue. Variant distribution did not significantly differ between schizophrenia and bipolar diagnoses (*P* > 0.4). Within the combined sample, the two variants were found to be highly linked from a linkage disequilibrium test (*D*′ = 0.92) using Haploview version 4.2 [36] and within a haplotype block using the conservative *four-gamete rule* (Figure 1). Pairwise haplotypes were calculated and empirical haplotype frequencies are presented in Table 3.

### 3.3. Clinical and Metabolic Characteristics according to Genotype and Haplotype

Table 4 represents the metabolic characteristics according to the *FADS1* genotype, *FADS2* genotype, and *FADS1/2 *haplotypes, respectively, for the combined sample. Significant differences are indicated within the table. Of the metabolic measurements, significant differences were identified with fasting insulin and HOMA-IR based on haplotype.

### 3.4. Regression Analysis of Lipid and Insulin Resistance Measures Based on Genotype or Haplotype

Our candidate gene hypotheses regarding the influence of *FADS1* and *FADS2* variants on lipid measures and insulin resistance were tested on the combined sample using generalized linear regression analysis adjusting for the following confounders: psychiatric diagnosis, age, gender, race, BMI, smoking status, and AAP status. This model was conducted for the *FADS1* dominant model, *FADS2* dominant model, and the inferred haplotypes. Due to multiple testing, only *P* values below 0.003 were considered statistically significant.

No statistically significant associations were found for the *FADS1* dominant model. An association was found for the *FADS2* dominant model with HOMA-IR (whole model *F*(9,301) = 6.77, *P* < 0.0001). However, this effect was mainly due to BMI (*P* < 0.0001) since the effect of *FADS2 *variant (*P* = 0.02) did not meet the more conservative cutoff value.

**Figure 2:**
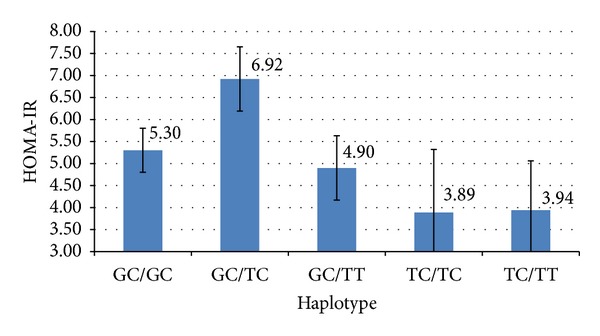
Association between HOMA-IR and *FADS* haplotype. Insulin resistance according to haplotype of *FADS1 *and *FADS2* polymorphisms adjusting for psychiatric diagnosis, age, gender, race, BMI, smoking status, and AAP use. Means ± s.e. bars.

When using haplotypes as the independent variable in our regression analysis, a trend with triglycerides was found that did not meet multiple testing cutoff (whole model *F*(12, 290) = 2.57, *P* = 0.003) due to the effect of BMI (*P* = 0.002) and haplotype (*P* = 0.004). Finally, of note, the significant association found above for HOMA-IR and haplotypes remained significant after controlling for confounders. The whole model was significant (*F*(12, 292) = 5.86, *P* < 0.0001) due to the effect of haplotype (*P* = 0.0004) and BMI (*P* < 0.0001). The HOMA-IR values for this significant association can be found in Figure 2.

## 4. Discussion

Within our study, we found that HOMA-IR was associated with the haplotype of two investigated *FADS* gene variants. This is the first report of such an association found in a population of schizophrenia and bipolar patients largely exposed to AAPs. However, associations with insulin measures have been reported previously in healthy Caucasian and Korean populations. Associations between *FADS1* polymorphisms and insulin resistance in a population with European descent arose from a GWAS meta-analysis conducted by Dupuis and colleagues [39]. This finding was subsequently confirmed via direct insulin resistance measures by Ingelsson and colleagues [40]. More recently, a study found correlations between HOMA-IR and variants in both the *FADS1* and *FADS2* genes in a population of healthy Korean men [41]. All of these studies used different polymorphisms found within the *FADS1 *and *FADS2* genes, and thus, our results reflect gene cluster associations previously found but not necessarily individual variant effects.

HapMap CEU data has shown that the *FADS1* gene and a large portion of the *FADS2* gene are known to be in a linkage disequilibrium block; therefore, haplotype analyses are a natural extension of *FADS* genetic investigations. However, there have been relatively few studies to date incorporating *FADS* haplotype in the analyses [32, 33, 42] which could be explained by the ability only to infer haplotypes when familial genetic data is unavailable. Haplotype reconstruction may be of particular importance in studies looking at insulin resistance measures or diabetes risk as D5D (*FADS1*) and D6D (*FADS2*) activities have been shown to have an opposite relationship with diabetes risk. Thus, examining polymorphisms from only one *FADS* gene region at a time does not take into account the opposing effects of the other *FADS* region. Our data demonstrates that *FADS1 *and *FADS2* variants had opposite relationships between HOMA-IR and the minor allele (nonsignificant, Table 4), but when the variants are combined into a haplotype, a relationship with HOMA-IR is exposed. This finding may indicate the importance of simultaneously considering variants from *FADS1 *and *FADS2* when exploring metabolic measures and particularly diabetes biomarkers in studies.

A nonsignificant trend after a multiple testing adjustment (*P* = 0.003) was identified for triglycerides and haplotype which mirrored the results of the statistically significant haplotype and HOMA-IR relationship. The same haplotype identified to have the highest HOMA-IR value, GC/TC, was found to have the highest TG level as well (147 mg/dL). The closest TG level was that of the GC/GC haplotype (116 mg/dL). This finding, although not statistically significant with the multiple testing correction, may be of clinical significance given the link between insulin resistance and TGs. Hypertriglyceridemia is considered a defining feature of insulin resistance and metabolic syndrome. TG-mediated changes in very-low-density lipoprotein and lipoprotein lipase activity ultimately lead to increased expression of angiopoietin-like protein 4, and thus, insulin resistance [43]. An association between the GC/TC haplotype and both HOMA-IR and triglyceride levels adds epidemiologic and mechanistic plausibility to our findings.

Several limitations of our study need to be addressed. First, this is a cross-sectional study and causal associations cannot be drawn from the data; prospective, randomized studies are needed in populations treated with AAPs to draw further conclusions. Second, our study had differences in demographics between the bipolar disorder and the schizophrenia subjects. While these are important differences to consider, we used psychiatric diagnosis as a confounder in our main hypothesis testing; therefore, these differences are a natural reflection of the diagnosis and recruitment area. Third, our subject population was largely exposed to AAPs with known metabolic side effects which makes translation of our results to other disease populations or populations exposed to other medications challenging. Fourth, we used a surrogate measure of insulin resistance, which, although not a direct measure of insulin resistance, has been highly correlated to more invasive measures such as the glucose clamps and the oral glucose tolerance test [25, 26]. HOMA-IR was used to make our findings more translatable to the clinic setting but more direct measures of insulin resistance would be useful in supporting our study's results. Our study did not collect dietary data or individually measure D5D and D6D fatty acid indices and these important measures should be taken into account in future studies. Finally, we only investigated one variant from each gene based on a literature search. Although this was done to strengthen our candidate gene approach, one cannot rule out the many polymorphisms found in this gene cluster including the *FADS1 *rs174550 variant, which has been previously implicated in insulin resistance.

Despite these limitations, our study is the first to identify a relationship between a *FADS1/2* haplotype and insulin resistance in a severely mentally ill population taking antipsychotics. Our population is unique in that it is at higher risk for insulin resistance and dyslipidemia due to the side effects associated with atypical antipsychotics. Indeed, those taking AAPs had higher HOMA-IR and lipid values compared to subjects taking typical antipsychotics (data not shown). AAP status was used as a confounder in our pharmacogenetic analysis; however, it adds evidence to the importance of discovering factors that may increase the risk for metabolic side effects including insulin resistance as this can lead to higher rates of cardiovascular disease and life years lost. The ability to personalize antipsychotic treatment based on a patient's metabolic risk profile, including pharmacogenetic analysis, may be one step to minimizing the damaging side effects of these drugs while maintaining their efficacy.

## 5. Conclusion

HOMA-IR was associated with a *FADS1/2* haplotype in a population with severe mental illness taking antipsychotics. This study is the first to identify such an association; however the results need to be repeated and further haplotype analyses with other high-risk *FADS* variants would substantiate the current findings.

## Figures and Tables

**Table 1:** Demographic and metabolic characteristics of the schizophrenia, bipolar, and combined samples.

	Schizophrenia spectrum (*n* = 226)	Bipolar disorder (*n* = 94)	Combined (*n* = 320)
Age (year)	42.7 ± 11.6	41.8 ± 12.0	42.5 ± 11.7
Gender (% female)	37	63	44.7^¥^
Race (% Caucasian/% African American/% other*)	65/26/9	81/12/7	70/21/9^¥^
Current smokers (%)	53	32	47^¥^
Currently on AAP (%)	85	74	82^¥^
BMI (kg/m^2^)	31.4 ± 7.24	31.8 ± 8.89	31.5 ± 7.74
SBP (mmHg)	122 ± 16.0	123 ± 17.7	122 ± 16.5
DBP (mmHg)	74.2 ± 11.4	72.9 ± 11.0	73.8 ± 11.3
TC (mg/dL)	179 ± 41.2	191 ± 45.7	183 ± 42.9^*¥*^
TG (mg/dL)	130 ± 88.9	142 ± 108	134 ± 95.0
HDL (mg/dL)	53.1 ± 17.3	57.6 ± 15.1	54.4 ± 16.8^*¥*^
LDL (mg/dL)	108 ± 34.8	118 ± 38.8	111 ± 36.2^*¥*^
Glucose (mg/dL)	99.0 ± 18.5	95.2 ± 10.7	97.9 ± 16.6
Insulin (*µ*IU/mL)	20.3 ± 14.6	24.3 ± 21.6	21.5 ± 17.0
HOMA-IR	5.07 ± 4.23	5.90 ± 5.66	5.32 ± 4.70

Means ± S.D. or percentage.

AAP: atypical antipsychotic, BMI: body mass index, SBP: systolic blood pressure, DBP: diastolic blood pressure, TC: total cholesterol, TG: triglycerides, HDL: high-density lipoprotein, LDL: low-density lipoprotein, and HOMA-IR: homeostasis model assessment-insulin resistance.

*Other includes self-defined race categories of Asian, Hispanic, latino, and others.

^*¥*^Significant difference based on a *P* value cutoff of 0.05.

**Table 2:** *FADS* genetic distribution for the schizophrenia, bipolar, and combined samples.

		Schizophrenia spectrum	Bipolar disorder	Combined
*FADS1* rs174537	GG genotype	54.5 (122)	48.9 (46)	52.8 (168)
	T allele	45.5 (102)	51.2 (58)	47.2 (150)
*FADS2* rs174570	CC genotype	75.2 (170)	71.3 (67)	74.1 (237)
	T allele	24.8 (56)	28.7 (27)	26.0 (83)

% (*n*); distribution based on dominant genetic model did not significantly differ between schizophrenia and bipolar diagnoses.

**Table 3:** Haplotype frequencies for combined sample.

Haplotype number	*FADS1* rs174537	*FADS2* rs174570	Frequency
1	G	C	0.708
2	G	T	0.006
3	T	C	0.159
4	T	T	0.127

Empirical haplotype frequencies. Gives total count for all haplotypes inferred.

**Table 4:** Demographic and metabolic characteristics based on *FADS* variants or *FADS* haplotype.

SNP/haplotypes	*FADS1* rs174537		*FADS2* rs174570		11	13	14	33	34
Genotype/allele/haplotype genotypes	GG genotype (*n* = 167)	T allele (*n* = 150)	CC genotype (*n* = 236)	T allele (*n* = 83)	GC/GC (*n* = 163)	GC/TC (*n* = 61)	GC/TT (*n* = 56)	TC/TC (*n* = 11)	TC/TT (*n* = 18)

Age (years)	42.8 ± 11.6	42.1 ± 11.9	43.2 ± 11.8	40.4 ± 11.2	43.0 ± 11.5	43.0 ± 11.6	39.3 ± 11.8	46.6 ± 16.7	44.3 ± 9.30
Gender (% female) ^*¥*#^	37	52	44	48	37	54	50	73	33
Race (% Caucasian/% African American/% other*)^*¥ψ*#^	57/34/9	83/8/9	65/27/8	81/8/11	57/35/8	82/10/8	78/11/11	100/0/0	94/6/0
Current smokers (%)	48	47	47	47	48	51	43	27	61
Currently on AAP (%)	78	85	81	84	78	88	85	91	73
BMI (kg/m^2^)^*ψ*^	31.3 ± 7.63	31.7 ± 7.91	31.0 ± 7.62	33.0 ± 7.92	31.4 ± 7.71	30.5 ± 7.61	33.5 ± 8.68	27.7 ± 5.99	31.2 ± 6.34
SBP (mmHg)	123 ± 16.1	121 ± 16.9	122 ± 16.4	123 ± 16.9	123 ± 15.9	119 ± 16.9	122 ± 14.4	121 ± 21.5	127 ± 21.7
DBP (mmHg)	74.3 ± 11.0	73.2 ± 11.6	74.0 ± 10.7	73.1 ± 12.7	74.4 ± 10.9	73.0 ± 10.7	73.2 ± 11.5	71.9 ± 6.95	76 ± 15.5
TC (mg/dL)	184 ± 41.7	181 ± 44.6	183 ± 43.1	182 ± 42.6	183 ± 41.3	184 ± 48.9	180 ± 41.6	181 ± 40.5	171 ± 42.3
TG (mg/dL)	131 ± 94.0	138 ± 96.4	136 ± 101.5	126 ± 72.1	130 ± 93.8	162 ± 124	121 ± 65.9	104 ± 45.6	127 ± 85.2
HDL (mg/dL)	54.0 ± 17.0	54.6 ± 16.3	54.4 ± 17.4	54.4 ± 15.1	54.0 ± 17.2	52.7 ± 16.0	55.2 ± 16.5	65.2 ± 20.3	51.8 ± 13.0
LDL (mg/dL)	112 ± 37.6	109 ± 34.9	110 ± 37.8	112 ± 31.5	111 ± 37.5	110 ± 39.9	110 ± 31.0	103 ± 32.5	105 ± 32.9
Glucose (mg/dL)	98.3 ± 17.9	97.2 ± 15.1	98.1 ± 17.1	97.2 ± 15.4	98.1 ± 18.0	98.6 ± 15.5	96.4 ± 16.2	94.2 ± 12.9	95.8 ± 12.4
Insulin (*µ*IU/mL)^#^	20.8 ± 12.3	22.4 ± 21.2	22.1 ± 18.4	19.7 ± 12.2	20.8 ± 12.3	27.3 ± 29.2	20.4 ± 13.9	15.7 ± 11.1	16.7 ± 6.51
HOMA-IR^#^	5.18 ± 3.74	5.49 ± 5.63	5.52 ± 5.13	4.72 ± 3.16	5.166 ± 3.74	6.90 ± 7.86	4.83 ± 3.54	3.89 ± 3.18	3.94 ± 1.61

*Other includes self-defined race categories of Asian, Hispanic, latino, and others.

^*¥*^Significant difference based on a *P* value cutoff of 0.05 for the *FADS1 *dominant genetic model.

^*ψ*^Significant difference based on a *P* value cutoff of 0.05 for the *FADS2* dominant genetic model.

^#^Significant difference based on a *P* value cutoff of 0.05 for haplotype comparisons.
